# Epidemiology and characteristics of urinary tract infections in children and adolescents

**DOI:** 10.3389/fcimb.2015.00045

**Published:** 2015-05-26

**Authors:** Rima H. Hanna-Wakim, Soha T. Ghanem, Mona W. El Helou, Sarah A. Khafaja, Rouba A. Shaker, Sara A. Hassan, Randa K. Saad, Carine P. Hedari, Rima W. Khinkarly, Farah M. Hajar, Marwan Bakhash, Dima El Karah, Imad S. Akel, Mariam A. Rajab, Mireille Khoury, Ghassan S. Dbaibo

**Affiliations:** ^1^Division of Pediatric Infectious Diseases, Department of Pediatrics and Adolescent Medicine, American University of BeirutBeirut, Lebanon; ^2^Center for Infectious Diseases Research, American University of BeirutBeirut, Lebanon; ^3^Department of Pediatrics, Makassed General HospitalBeirut, Lebanon; ^4^Department of Biochemistry and Molecular Genetics, Faculty of Medicine, American University of BeirutBeirut, Lebanon

**Keywords:** ESBL, urinary tract infection, risk factors, children, antibiotic resistance

## Abstract

**Background:** Urinary tract infections (UTIs) are among the most common infections in the pediatric population. Over the last two decades, antibiotic resistance is increasing significantly as extended spectrum beta lactamase (ESBL) producing organisms are emerging. The aim of this study is to provide a comprehensive view of the epidemiologic characteristics of UTIs in hospitalized children, examine the risk factors of UTIs caused by ESBL-producing organisms, and determine the resistance patterns in the isolated organisms over the last 10 years.

**Methods:** Retrospective chart review was conducted at two Lebanese medical centers. Subjects were identified by looking at the following ICD-9 discharge codes: “Urinary tract infection,” “UTI,” “Cystitis,” and/or “Pyelonephritis.” Children less than 18 years of age admitted for UTI between January 1st, 2001 and December 31st, 2011 were included. Cases whose urine culture result did not meet our definition for UTI were excluded. Chi-square, Fisher's exact test, and multivariate logistic regression were used to determine risk factors for ESBL. Linear regression analysis was used to determine resistance patterns.

**Results:** The study included 675 cases with a median age of 16 months and female predominance of 77.7% (525 cases). Of the 584 cases caused *by Escherichia coli* or *Klebsiella* spp, 91 cases (15.5%) were found to be ESBL-producing organisms. Vesico-ureteral reflux and previous antibiotics use were found to be independent risk factors for ESBL-producing *E. coli* and *Klebsiella* spp. (*p* < 0.05). A significant linear increase in resistance to all generations of Cephalosporins (*r*^2^ = 0.442) and Fluoroquinolones (*r*^2^ = 0.698) was found.

**Conclusion:** The recognition of risk factors for infection with ESBL-producing organisms and the observation of increasing overall resistance to antibiotics warrant further studies that might probably lead to new recommendations to guide management of UTIs and antibiotic use in children and adolescents.

## Introduction

Urinary tract infections (UTIs) are among the most commonly encountered infections in the pediatric age group both in the community and hospital settings (Ronald et al., 2001; Stamm and Norrby, 2001; Nicolle, 2002; Hooton et al., 2004). Several studies from the United States of America estimate the direct and indirect cost of acute pyelonephritis in adults to 2.14 billion US dollars (year 2000 values) which is 2.19 billion US dollars in 2013 (Brown et al., 2005; Foxman, 2014). It is estimated that 150 million UTIs occur yearly worldwide, resulting in more than 6 billion dollars in direct healthcare cost (Stamm and Norrby, 2001).

Over the last two decades, the resistance to antibiotics in members of Gram-negative Enterobacteriaceae rose tremendously worldwide; highlighted by the emergence of extended spectrum beta-lactamase (ESBL) producing organisms (Ena et al., 2006; Pitout and Laupland, 2008; Ben-Ami et al., 2009; Soraas et al., 2013). Whereas the initial spread was in hospital settings, eventually these pathogens emerged in community-onset UTIs (Pitout and Laupland, 2008). In many countries of the Middle East, where unregulated prescription of antibiotics is prevalent, the resistance patterns of frequently observed uropathogens are alarming (Kanafani et al., 2005; Topaloglu et al., 2010; Daoud and Afif, 2011; Araj et al., 2012; Kizilca et al., 2012; Pourakbari et al., 2012; Al-Assil et al., 2013; Dayan et al., 2013; Dotis et al., 2013; Megged, 2014). However, there is limited data concerning the antibiotic resistance pattern of pediatric UTIs in the region of the Middle East.

Few studies have investigated the prevalence and risk factors associated with hospital and community-acquired UTIs secondary to ESBL-producing bacteria in the pediatric age group.

Previous studies have shown that renal abnormalities, septicemia, systemic disease such as metabolic diseases and malignancies, hospitalization within the previous 3 months preceding the onset of UTI, age less than 1 year, high recurrence rate of UTI, and recent antibiotic treatment are independent risk factors for UTI secondary to community-acquired ESBL-producing organisms (Topaloglu et al., 2010; Kizilca et al., 2012; Dayan et al., 2013; Dotis et al., 2013; Giardino et al., 2013; Megged, 2014). Those risk factors have not been well established in our region.

Accordingly, the purpose of this retrospective study is to provide a comprehensive review of the epidemiology and characteristics of UTIs in hospitalized children and adolescents, determine the risk factors of UTIs caused by ESBL-producing organisms of *E. coli* and *Klebsiella* spp, and examine the resistance patterns in the isolated organisms over the last 10 years.

## Materials and methods

### Study design

A multi-center retrospective cohort study was conducted in two major Lebanese hospitals both in Beirut; the American University of Beirut Medical Center (AUBMC) and Makassed General Hospital (MGH). The study was approved by the institutional review board (IRB) at each center (the AUB IRB approval number was PED.RW.01, the MGH IRB approval number was dated 12 June 2013).

All patients were identified retrospectively through medical records at AUBMC and MGH by looking at the following ICD-9 codes for discharge diagnosis: “Urinary tract infection,” “UTI,” “Cystitis,” and/or “Pyelonephritis.” Children less than 18 years of age with one of the above discharge diagnosis, admitted to the hospital between January 1st, 2001 and December 31st, 2011 were included in the study. We excluded patients whose urine culture result did not meet the definition for UTI established according to the clinical practice guidelines issued in 2011 for the diagnosis and management of UTI (Roberts, 2011). Accordingly, this included significant bacteriuria with recovery of at least 100,000 CFU/mL (colony forming unit per milliliter) of a single uropathogen from a clean catch specimen; or at least 50,000 CFU/ mL of a single uropathogen from a catheterized specimen; or any uropathogenic bacteria from a suprapubic aspirate.

### Data collection

Data from each reviewed chart was documented on a case report form (CRF). Data collected included the following information: basic demographics (age, gender), hospital stay information (ICU/regular floor admission, length of stay), past medical history (underlying diseases, genitourinary disorders, immunosuppression), previous antibiotic use, recent hospitalizations, past surgical history, method of urine collection, laboratory information (urinalysis, urine culture, antimicrobial susceptibility patterns, complete blood count, basic metabolic panel), imaging results, clinical course and outcomes.

### Analysis and reporting of results

The Statistical Package for Social Sciences (SPSS) program, version 22.0 for Windows was used for data analysis (IBM, Armonk, NY). Simple descriptive statistics were used to describe patients' demographics and characteristics of UTI. Bivariate and multivariate analyses of risk factors for ESBL were first analyzed by Pearson's Chi-square test or Fisher's exact test (when number of subjects in a subgroup was less than 5). Continuous risk factors were analyzed with student *t*-test. Statistical significance was considered below a type-1 error threshold (alpha level) of 0.05. Following that a multivariate logistic regression model comprised of significant risk factors was constructed and reported.

Linear regression analysis was conducted to determine resistance patterns to different classes of antibiotics over the past 10 years (correlation coefficient *r*^2^ and *p*-value for regression analysis were reported).

## Results

### Number of cases

**Figure 1:**
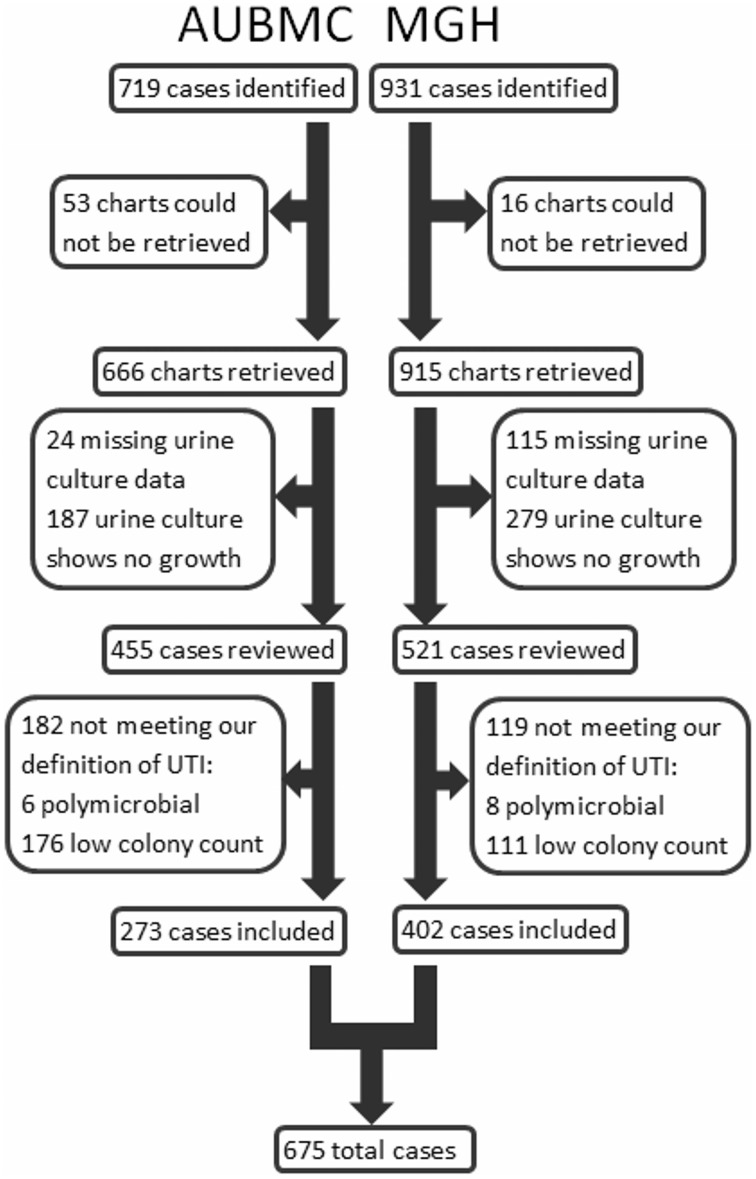
**Identification and inclusion of cases into the study**.

Cases were identified and included in our study as shown in Figure 1. Our search retrieved 1650 cases: 719 at AUBMC and 931 at MGH. Only 666 records at AUBMC and 915 records at MGH were available for review. Of these, 211 cases at AUBMC and 394 cases at MGH were excluded for either having missing urine culture data or a negative urine culture result (no growth). The remaining cases were reviewed. Of these, 182 cases at AUBMC and 119 cases at MGH did not meet our definition of UTI and were excluded from the study. Therefore, a total of 675 cases were included in the analysis.

### Demographic characteristics

Our study population was divided into three groups, (less than 2 months, between 2 months and 2 years, and more than 2 years), which had a distribution of 9.5, 49.6, and 40.9% respectively. Overall, females were predominant (77.7%). However, in infants less than 2 months, 60.9% were males (Table 1). The median age was 16 months. The majority of males were circumcised (66%).

**Table 1:** **Gender distribution by age groups**.

**Gender**	**Age group *n* (%)**			**Total**
	**<2 months**	**2 months–2 years**	**>2 years**	
Females	25 (39.1)	254 (75.8)	246 (89.1)	525 (77.7)
Males	39 (60.9)	81 (24.2)	30 (10.9)	150 (22.3)
Total	64 (9.5)	335 (49.6)	276 (40.9)	675

### Isolated microorganisms

*E. coli* was the most common pathogen isolated among all age groups, followed by Klebsiella and Proteus species, accounting for 79.4, 7.9 and 3.9% respectively. Other organisms such as *Pseudomonas, Enterococcus, and Enterobacter* were relatively rare (Table 2).

**Table 2:** **Isolated uropathogens by age groups**.

**Organisms**	**Total *n* (%)**		**Age groups *n* (%)**	
		**<2 months**	**2 months –2 years**	**>2 years**
*E. coli*	536 (79.4)	39 (60.9)	266 (79.4)	231 (83.7)
*Klebsiella* spp.	53 (7.8)	18 (28.1)	26 (7.7)	9 (3.3)
*Proteus* spp.	26 (3.8)	1 (1.6)	12 (3.6)	13 (4.7)
*Pseudomonas aeruginosa*	14 (2.1)	1 (1.6)	5 (1.5)	8 (2.9)
Enterococcus spp.	14 (2.1)	0 (0)	8 (2.4)	6 (2.2)
Enterobacter spp.	10 (1.5)	2 (3.1)	6 (1.8)	2 (0.7)
Others^*^	22 (3.3)	3 (4.7)	12 (3.6)	7 (2.5)
Total	675 (100)	64 (100)	335 (100)	276 (100)

* *Others: Candida albicans, Candida non-albicans, coagulase negative Staphylococcus, Group B Streptococcus, Providencia startii, Citrobacter freundii, viridans group streptococci, Serratia marcescens, Morganella morgani, Alcaligenes faecalis, Citrobacter diversus, Salmonella group C*.

### Imaging and laboratory findings

**Figure 2:**
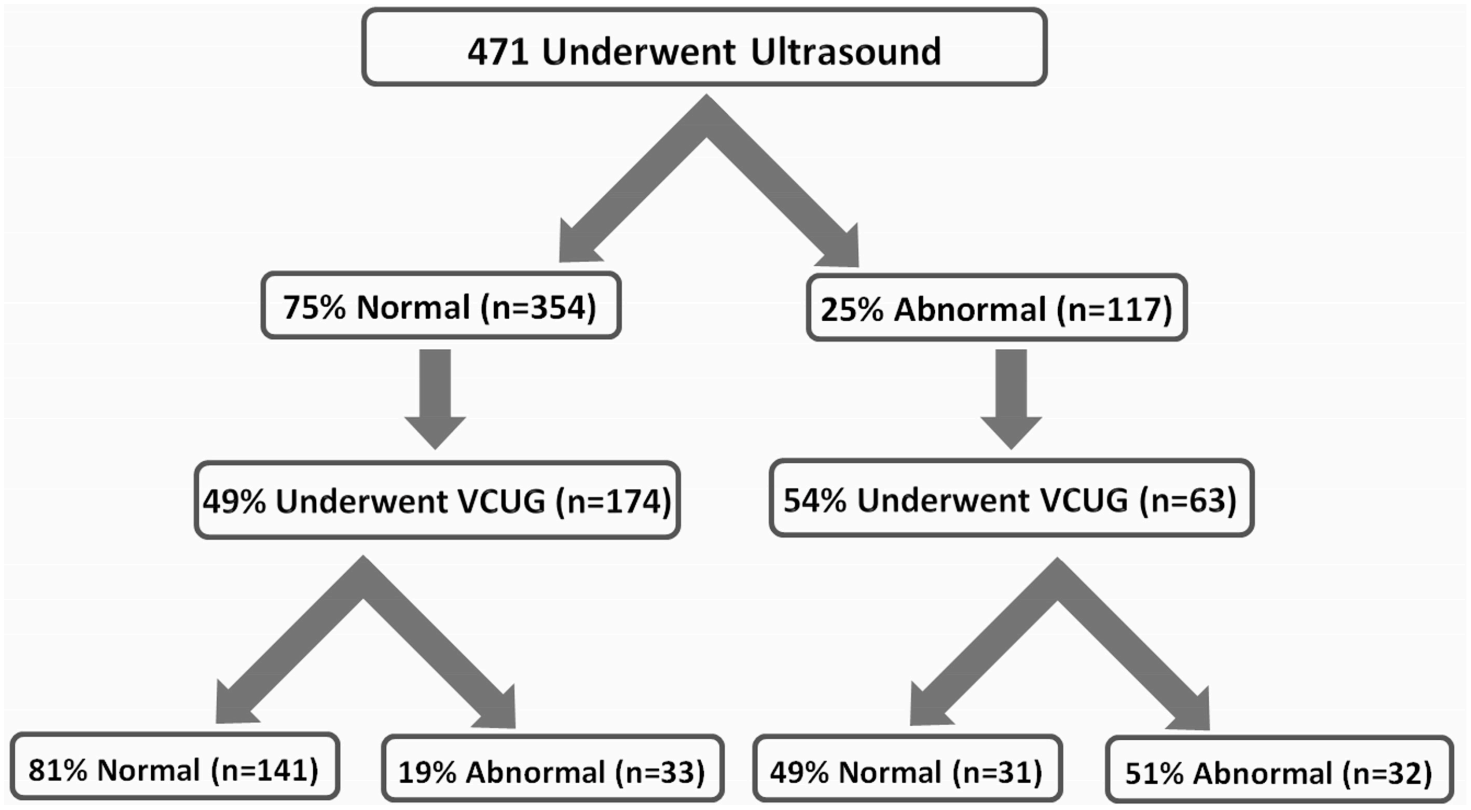
**Imaging results for ultrasound and voiding cystourethrogram (VCUG)**.

Renal ultrasound was performed in 69.8% of cases, 24.8% of the results were abnormal. Dilation of the pelvicalyceal system was the most common finding, representing 41.9% of abnormalities. Voiding cystourethrogram (VCUG) was performed in 53.4% of the cases with an abnormal ultrasound. Vesicoureteral reflux (VUR) was identified in 51% of these cases. In addition, 49.3% of the cases with normal ultrasound findings underwent VCUG, of which 19% had VUR (Figure 2).

Out of 273 cases from AUBMC, 103 underwent Dimercaptosuccinic acid (DMSA) radio-nucleotide scans, of which 60 cases had scarring or foci of pyelonephritis. No DMSA data was reported from MGH, as this procedure was not available in this hospital. When comparing ESBL and non-ESBL-producing *E. coli* and *Klebsiella* spp, 25.5% of cases with abnormal DMSA results had ESBL positive UTI.

Urinalysis showed a high white blood cell (WBC) count of more than 5 per high-power field (HPF) in only 60.1% of cases. Leukocyte esterase and nitrites were positive in only 80.2 and 39% of our cases, respectively. Approximately one third of patients (34.9%) had all three positive findings present on urinalysis.

### ESBL risk factors and emergence patterns

The ESBL-producing and non-ESBL-producing groups consisted of 91 (15.5%) and 493 (84.5%) cases, respectively. ESBL cases were significantly more frequent at AUBMC (*p* < 0.05). For 5 of the 589 cases caused by *E. coli* or *Klebsiella* spp, the data on ESBL status was missing.

Median age, gender, and age group distribution revealed no significant differences between the ESBL-producing and non-ESBL-producing groups. In the ESBL-producing group, *E. coli* and *Klebsiella* isolates represented 82.4% and 17.6%, respectively; whereas, in the non-ESBL-producing group, *E. coli* and *Klebsiella* isolates represented 92.5 and 7.5%, respectively. Thus, *Klebsiella* spp. were more commonly found in the ESBL-producing group (*p* = 0.02). Additionally, longer duration of hospital stay was found to be statistically significant in cases with ESBL-producing organisms (*p* < 0.001) (Table 3).

**Table 3:** **Characteristics of ESBL and Non-ESBL groups**.

	**ESBL (*N* = 91)**	**Non-ESBL (*N* = 493)**	***p*-value^†^**
Median age in months	23	15	
Mean age in months	38.8	38	0.883^*^
Female Gender, *n* (%)	77 (84.6)	395 (80.2)	0.318
**AGE GROUPS, *N* (%)**			
<2 months	8 (8.8)	48 (9.7)	0.368
2 months–2 years	40 (44)	251 (51)	
>2 years	43 (47.2)	194 (39.3)	
**PREVALENCE BY LOCATION (%)**			
MGH	12.1	87.9	0.004
AUBMC	21.1	78.9	
**ORGANISMS, *N* (%)**			
*E. coli*	75 (82.4)	456 (92.5)	0.020
*Klebsiella* spp.	16 (17.6)	37 (7.5)	
**Mean LOS**	14.33	6.48	<0.001^*^

*LOS Length of hospital stay in days*.

* *Independent Sample-t test is used to compare means*.

† *Pearson's Chi-Square test was used (no expected count less than 5)*.

### Clinical characteristics

When comparing ESBL and non-ESBL groups, there was no significant difference in the presenting symptoms regarding fever, vomiting, diarrhea, irritability, abdominal pain, flank pain and voiding dysfunction. However, enuresis was significantly higher in the ESBL group (*p* < 0.05).

Although liver, renal, and cardiac diseases were more prevalent in ESBL cases, the difference was not statistically significant (*p* > 0.05). On the other hand, VUR, recurrent UTI, and history of genitourinary (GU) surgery were found to be significantly higher in ESBL group (*p* < 0.05). Regarding circumcision, no significant difference was observed (*p* = 0.703) (Table 4).

**Table 4:** **Possible risk factors for ESBL cases**.

**Risk factors**	**ESBL *n* (%)**	**Non-ESBL *n* (%)**	***p*-value^†^**
Systemic diseases^**^	4 (4.4)	18 (3.7)	0.763^*^
Renal disease	8 (8.8)	20 (4.1)	0.062^*^
PUV	3 (3.3)	1 (0.2)	0.013^*^
VUR	13 (14.3)	14 (2.8)	<0.001^*^
Recurrent UTI	36 (39.6)	76 (15.4)	**<0.001**
GU surgery	13 (14.3)	15 (3)	<0.001^*^
Circumcision	10 (76.9)	69 (82.1)	0.703^*^
Urinary catheter	7 (7.7)	13 (2.6)	0.024^*^
Non-GU surgery	18 (19.8)	48 (9.7)	**0.005**
Previous antibiotic use	34 (37.4)	46 (9.3)	**<0.001**
Use of suppressive antibiotic	15 (16.5)	12 (2.4)	<0.001^*^
Previous hospitalization	19 (20.9)	26 (5.3)	**<0.001**
Immunocompromised^***^	12 (13.2)	22 (4.5)	**0.001**
Chemotherapy	11 (12.1)	7 (1.4)	<0.001^*^
Radiation therapy	3 (3.3)	3 (0.6)	0.051^*^
Diarrhea in the preceding week	15 (16.5)	94 (19.1)	0.561
Constipation	6 (6.6)	21 (4.3)	0.411
Toilet training: urine	28 (30.8)	156 (31.6)	0.869
Toilet training: stool	29 (31.9)	157 (31.8)	0.997
CVC or Arterial line	14 (15.4)	20 (4.1)	**<0.001**
ETT	2 (2.2)	4 (0.8)	0.237^*^

*PUV, Posterior urethral valve; CVC, Central venous catheter; ETT, Endotracheal tube*.

*Significant differences for p-values are indicated in bold*.

† *Pearson Chi-Square was used (no expected count less than 5)*.

* *Fisher's exact test was used (at least one expected count less than 5)*.

** *Systemic Diseases: Cardiac disease, liver disease, pulmonary disease and diabetes*.

*** *Primary or secondary to malignancy (lymphoma, leukemia, or solid tumor)*.

A multivariate logistic regression analysis was performed with the above risk factors while controlling for age and gender. VUR and previous antibiotic use were found to be independent risk factors with odds ratio (OR) of 3.01 and 4.05, respectively.

### Resistance to different classes of antibiotics

**Figure 3:**
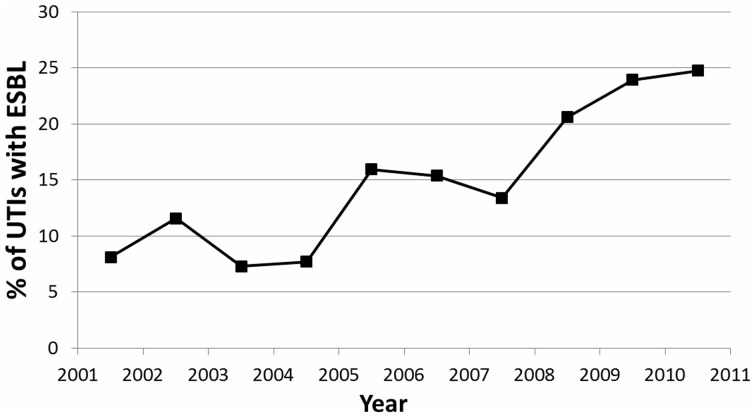
**Trend showing percentage of UTIs caused by ESBL-producing organisms over the 10 year period of the study**.

When a linear regression was performed comparing ESBL pattern over the past 10 years, a significant association was found (*p* = 0.004) (Figure 3). There was a strong positive linear correlation (*r*^2^ = 0.783), with a 2.08% yearly increase in UTIs secondary to ESBL-producing bacteria.

To observe the trends in UTI resistance to different classes of antibiotics in the period of 2001 to 2011, we plotted the yearly percentage of UTIs resistant to gentamicin, cephalosporins, fluoroquinolones, and trimethoprim-sulfamethoxazole (TMP-SMX). Resistance to first generation cephalosporins could not be evaluated because it is not tested for by the hospitals' laboratories included in this study. Since the trends in antibiotic resistance to second, third, and fourth generations of cephalosporins were very similar, we grouped the three generations of cephalosporins into one class. The fluoroquinolone class of antibiotics in our study included levofloxacin and ciprofloxacin; resistance to other fluoroquinolones is not tested by the hospitals' laboratories included in this study. The trends in antibiotic resistance to levofloxacin and ciprofloxacin were also similar.

**Figure 4:**
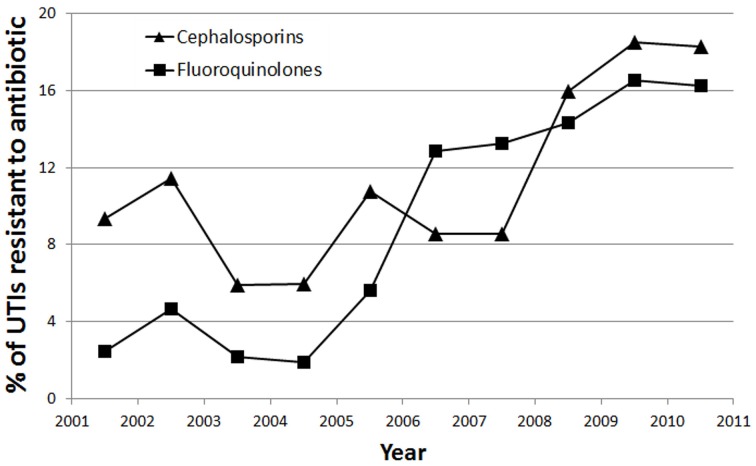
**Trend showing percentage of UTIs resistant to Cephalosporins (▲) and Fluoroquinolones (■) over the 10 year period of the study**.

Linear regression revealed statistically significant linear trends in antibiotic resistance to fluoroquinolones (*p* = 0.001) and cephalosporins (*p* = 0.026). No significant linear association was found for resistance to penicillin derivatives (*p* = 0.09), amikacin (*p* = 0.444), TMP-SMX (*p* = 0.684), and gentamicin (*p* = 0.065) across the years 2001–2011. Significant trends plotted are shown in Figure 4.

There was a strong linear association for resistance to fluoroquinolones across time (*r*^2^ = 0.698), such that there was a 1.62% yearly increase in UTIs resistant to fluoroquinolones. There was a moderate linear association for resistance to cephalosporins across time (*r*^2^ = 0.442), such that there was a 1.21% yearly increase in UTIs resistant to cephalosporins.

### Antibiotic usage

The most common class of antibiotics used for inpatient treatment of UTIs in our study was cephalosporins, with 76.9% of patients with UTIs having used at least one cephalosporin during inpatient treatment. The most commonly used cephalosporins were third generation cephalosporins, used in 67.5% of cases. Second generation cephalosporins were used in 7.1% of cases, whereas fourth generation cephalosporins were used in 2.5% of cases. A penicillin derivative, most commonly ampicillin or amoxicillin-clavulanic acid, were used for inpatient treatment in 16% of cases. Aminoglycosides (amikacin or gentamicin) were used in 12.3% of cases. Carbapenems, glycopeptides, and fluroquinolones were used in 10.5, 3.3, and 1% of cases respectively. Simultaneous use of two or more antibiotics was practiced in 10.1% of cases; whereas 22.2% of cases required sequential use of multiple antibiotics.

Regarding antibiotic use on discharge, 66.2% of cases were discharged on at least one antibiotic with oral third generation cephalosporins used in 43.65% of cases.

## Discussion

### Epidemiologic characteristics of UTI

UTIs are among the most frequent infections encountered in the pediatric population. To our knowledge this is the first Lebanese retrospective pediatric study that reviews the epidemiology of UTIs in hospitalized children, and outlines the risk factors and outcomes of UTIs caused by ESBL-producing *E. coli* and *Klebsiella* spp.

The epidemiology of pediatric UTIs depends on several factors such as age, gender, and genitourinary malformations. Our study showed an overall female predominance; this could be related to circumcision as 78 % of males in our study were circumcised. This is presumed to be the result of geographic location and religious affiliation. In addition, the shorter female urethra is a plausible explanation of the increased prevalence of UTIs in females. Zorc et al. mentioned that UTI is prevalent in all children in their first year but after 1 year of age, it tends to decrease in males due to multiple factors such as circumcision (Circumcision, 1999).

Approximately, one-fifth of our patients (19.7%) with normal renal ultrasound had VUR, with approximately one-fourth (24.1%) being grade V. These findings were similar in comparison with other studies. Juliano et al. reported that despite normal renal bladder ultrasound, 24% of their patients had dilating vesicoureteral reflux (Juliano et al., 2013). Another study showed that the sensitivity of renal ultrasound in suggesting VUR was only 40% (Mahant et al., 2002). DiPietro et al found that renal ultrasound was unreliable in excluding VUR in children aged 5 years or older who were being evaluated for a UTI (Dipietro et al., 1997). Our study showed that approximately 5% of the patients who had normal renal ultrasound had VUR grade V needing surgical management, which means if VCUG is deferred, according to the last published guidelines in 2011(Roberts, 2011), parents should be counseled regarding this risk. Thus, if our findings are confirmed in other similar studies, the guidelines may have to be revisited.

The absence of pyuria in children with UTIs was found in a high percentage of cases (40%). This discordance was higher than that reported in previous studies (27%) (Roberts, 2011). There was no significant difference in the presence of pyuria between immunocompromised and non-immunocompromised patients (64.5% of immunocompromised patients had positive urinalysis WBC compared to 68% of non-immunocompromised patients). Nitrites were positive in only 39% of cases of UTI documented by urine culture. This is lower than the reported sensitivity in previous studies (53%) (Roberts, 2011). Leucocyte esterase was positive in 80.2% of cases of UTI documented by urine culture. This is similar to the reported sensitivity of leucocyte esterase in various studies (83%) (Roberts, 2011), making it a reliable test for predicting UTIs in young febrile children.

### Risk factors for ESBL

Our study revealed that *E. coli* is the most common pathogen in UTIs among all age groups, whether ESBL or non-ESBL producers. This finding is compatible with other studies (Calbo et al., 2006; Topaloglu et al., 2010; Kizilca et al., 2012; Dotis et al., 2013). Among *E. coli* and *Klebsiella* spp, the proportion of ESBL-producing bacteria was higher in *Klebsiella* spp. This was also reported by Kiziliza et al. suggesting that if the isolated microorganism of UTI belongs to *Klebsiella* spp, production of ESBL may be expected more frequently (Kizilca et al., 2012).

Lautenbach et al. revealed no association between hospital location and infection with ESBL-producing *E. coli* or *K. pneumonia* (Lautenbach et al., 2001). However, in our study, we found that ESBL infection is more common at AUBMC than MGH. This can be attributed to notable differences between the two hospitals. AUBMC is Lebanon's largest medical center, and has referral units for pediatric cardiology, oncology, neurosurgery and intensive care patients. Although both hospitals are tertiary care centers, MGH is a smaller center with fewer facilities than AUBMC. As such, the cases referred to AUBMC tend to be more complicated than cases admitted to MGH. As for the association with immunosuppression: immunosuppression secondary to malignancy and chemotherapy places the patient at higher risk of acquiring resistant bacteria (as ESBL producing bacteria) due to the multiple visits/stays in hospitals and the use of broad spectrum antibiotics.

Except for enuresis, there were no statistically significant differences in the presenting symptoms between ESBL and non-ESBL UTI. This is in contrast to a previous study (Dotis et al., 2013) that showed children with ESBL UTI presented clinically with more symptoms than children with non-ESBL UTI. In fact, our results indicate that UTIs secondary to ESBL-producing organisms are not associated with a more severe clinical picture.

Some of the risk factors for ESBL UTI that our study showed to be significant as recurrent UTIs, previous urological abnormalities, previous antibiotic use, previous hospitalization, and malignancies were found to be significant in other studies (Lautenbach et al., 2001; Topaloglu et al., 2010; Kizilca et al., 2012; Megged, 2014). However, only previous antibiotic use was found to be an independent risk factor. This is in contrast to the study by Topaloglu et al. (2010), that found previous antibiotic use was not an independent risk factor.

The mean length of hospital stay was significantly longer for patients with UTI caused by ESBL-producing bacteria than that for patients with UTI caused by non-ESBL-producing bacteria. This may be attributed to the fact that ESBL treatment in children necessitates parenteral antibiotic administration for 7–10 days; as opposed to adults, where oral Ciprofloxacin is a treatment option for ESBL UTI. Other studies also found that longer hospitalization periods are needed for the treatment of ESBL infections (Lautenbach et al., 2001; Megged, 2014). As quinolones become more routinely used in children and adolescents, this trend for longer hospitalizations with ESBL infections may be reversed but the rising quinolone resistance might cancel this hope.

### Resistance trends and usage of different classes of antibiotics

In our study over a 10-year period, there was an alarming increase in the incidence of ESBL-producing UTIs in hospitalized children, from 8% in 2001 to 25% in 2011 (Figure 3). A Lebanese study by Araj et al. on bacterial susceptibility patterns in all infections among all ages found nearly similar results within the same time period (Araj et al., 2012). Araj et al. demonstrated an increase of ESBL-producing *E. coli* and *Klebsiella* spp, from 4 and 12% in 2000, to 30 and 28% in 2011, respectively (Araj et al., 2012). A comparable finding was also demonstrated at another center in Lebanon by Daoud et al. (Daoud and Afif, 2011), who observed an increase in UTIs caused by ESBL-producing organisms from 2.3% in 2000 to 16.8% in 2009 among patients of all ages. Both studies attributed this finding to uncontrolled antimicrobial usage in Lebanon (Daoud and Afif, 2011; Araj et al., 2012).

Although percentage of UTIs resistant to TMP-SMX was considerably more than resistance to any other antibiotic, there has been no increase in resistance to TMP-SMX across the years. One study in the United States showed that TMP-SMX is a poor empirical choice in many areas due to high resistance rates (Edlin et al., 2013). Our study revealed that in our setting, TMP-SMX still has a role in the treatment of UTIs; however it is usually reserved for use upon discharge and is infrequently used as inpatient treatment for hospitalized patients.

The trend in increasing resistance to fluoroquinolones has been well established in other studies. A study examining the epidemiology and resistance in uncomplicated UTI among adult females in Europe and Brazil found a significant increase in quinolone resistance of community-acquired urinary *E. coli* (Schito et al., 2009). One review attributed this trend in increasing fluoroquinolone resistance to overuse of this class of antibiotics (Nickel, 2007). While the results on antibiotic usage within our cohort reveal relatively controlled usage of fluoroquinolones, one must keep in mind that our results only shed light on antibiotic practice in treating UTI in hospitalized children. It is likely that there is in fact overuse of fluoroquinolones in our population's healthcare system, especially among adults in outpatient settings, leading to community-acquired resistance. In addition, as a study conducted in Canada has shown (Pepin et al., 2009), risk factors for the development of UTIs resistant to ciprofloxacin included the use of aminoglycosides in the last 12 months. Therefore, the trend of increasing resistance of uropathogens to fluoroquinolones may also be due to cross-reactivity from overuse of other antibiotics.

We also observed a significant trend of increasing resistance to cephalosporins over the study's 10 year period (Figure 4). This is likely due to overuse of antibiotics in our community, as our study has shown that the majority (76.9%) of hospitalized UTIs were treated with cephalosporins, specifically third generation cephalosporins. Our dependence on cephalosporins in light of increasing resistance will pose a challenge in the future, and emphasizes the need to find alternative antibiotics.

### Limitations

Although our study is the first to investigate the epidemiology of UTIs and risk factors for ESBL in the pediatric population in Lebanon, it is important to note certain limitations. Our study only looked at hospitalized children with UTI, and therefore our findings may not apply to the general pediatric population. Furthermore, the number of cases included in our study was restricted by our criteria for defining UTI. A significant number of cases excluded due to negative or low colony count on urine culture are likely the result of over-the-counter antibiotic use before the culture was taken. A study examining the value of semi-quantitative bacterial counts in establishing the diagnosis of UTI caused by group B streptococcus in adults demonstrated occurrence of UTI at values as low as 100 CFU/ml (Tan et al., 2012). Similarly, in abiding by the cutoffs mentioned in our study we may have missed a substantial number of UTI cases with subthreshold colony counts. Finally, our study is not immune to the inherent limitations of a retrospective chart review; the cases included in our study were limited by the availability of data and incomplete medical records, such as missing laboratory results for urine cultures or other studies that were done in outside hospitals.

## Conclusion

In conclusion, the resistance to commonly used antibiotics for UTIs has been increasing over the last 10 years, as ESBL-producing organisms are emerging. The recognition of the epidemiology and risk factors for ESBL-producing bacteria in the pediatric population may affect our management and therapeutic approach. In keeping with our finding that previous antibiotic use was the most important independent risk factor for emergence of ESBL-producing bacteria, further studies and new recommendations that guide management of UTIs and antibiotic use are warranted.

### Conflict of interest statement

The authors declare that the research was conducted in the absence of any commercial or financial relationships that could be construed as a potential conflict of interest.
